# Bioinformatics analysis of the role of CXC ligands in the microenvironment of head and neck tumor

**DOI:** 10.18632/aging.203269

**Published:** 2021-07-11

**Authors:** Fengyang Jing, Jianxiong Wang, Liming Zhou, Yujie Ning, Shengqian Xu, Youming Zhu

**Affiliations:** 1 Department of Dental Implant Center, Stomatologic Hospital and College, Anhui Medical University, Key Laboratory of Oral Diseases Research of Anhui Province, Hefei 230032, China; 2 Chief Physician, Department of Rheumatology and Immunology, The First Affiliated Hospital of Anhui Medical University, Hefei 230022, China

**Keywords:** head and neck carcinoma, bioinformatics analysis, tumor immunology, CXC ligand, biomarker

## Abstract

Chemokines play a significant role in cancer. CXC-motif chemokine ligands (CXCLs) are associated with the tumorigenesis and progression of head and neck squamous cell carcinoma (HNSC); however, their specific functions in the tumor microenvironment remain unclear. Here, we analyzed the molecular networks and transcriptional data of HNSC patients from the Oncomine, GEPIA, String, cBioPortal, Metascape, TISCH, and TIMER databases. To verify immune functions of CXCLs, their expression was analyzed in different immune cell types. To our knowledge, this is the first report on the correlation between CXCL9–12 and 14 expression and advanced tumor stage. CXCL2, 3, 8, 10, 13, and 16 were remarkably related to tumor immunity. Kaplan–Meier and TIMER survival analyses revealed that high expression of CXCL1, 2, 4, and 6–8 is correlated with low survival in HNSC patients, whereas high expression of CXCL9, 10, 13, 14, and 17 predicts high survival. Only CXCL13 and 14 were associated with overall survival in human papilloma virus (HPV)-negative patients. Single-cell datasets confirmed that CXCLs are associated with HNSC-related immune cells. Thus, CXCL1–6, 8–10, 12–14, and 17 could be prognostic targets for HNSC, and CXCL13 and 14 could be novel biomarkers of HPV-negative HNSC.

## INTRODUCTION

Head and neck squamous cell carcinoma (HNSC) constitutes 5.7% of cancer-related mortality worldwide [1]. Lymph node metastasis is known to reduce the cancer-specific survival of HNSC patients by approximately 50% [2], while extranodal extension (ENE) increases regional and distant metastatic failure [3]. However, the pathogenicity of local tumor cell implantation during primary surgical intervention and the effects of wound healing growth factors on HNSC proliferation remain unclear [4]. Although human papilloma virus (HPV) infection is currently the only molecular biomarker available to individualize treatment options for oropharyngeal cancer, recent studies have elucidated biological interactions within the tumor microenvironment [5, 6].

Chemokines are a small class of cytokine-like molecules with four subtypes: C-X-C (CXC), C-C (CC), C-X3-C (CX3C), and X-C (XC), where X represents a conserved terminal cysteine residue [7]. Various CXC-motif chemokine ligands (CXCLs), termed CXCL1–17, have been identified to date [8]; all of these, except CXCL15, are found in humans (Supplementary Table 1). Chemokines indirectly participate in tumor development by affecting angiogenesis and the interaction between tumors and leukocytes, as well as by directly affecting tumor transformation, survival, growth, invasion, and metastasis. Although chemokines can kill tumors by regulating white blood cell infiltration and activating an immune response [9, 10], they can also promote tumor tissue angiogenesis, accelerate tumor cell proliferation, and promote basement membrane invasion, thereby promoting tumor growth and metastasis [11–13].

Several studies have shown that CXCLs may individually or synergistically play complex and distinct roles in HNSC by affecting tumor cell proliferation, migration, invasion, growth, and survival via various pathways to modulate tumor growth and metastasis [14–28]. Although these studies have elucidated the roles of different CXCLs in various oral cancers, the potential activation or inhibition mechanisms and functions of CXCLs in the HNSC tumor microenvironment have not yet been fully elucidated.

DNA and RNA sequencing techniques are important aspects of biological and biomedical research that have been revolutionized by the development of microarray technology; however, the roles of CXCLs in HNSC have not yet been analyzed using bioinformatic methods, and the immune functions of CXCLs in HNSC remain unclear. Based on published gene expression and copy number variation datasets, we analyzed the expression of CXCLs in HNSC patients to determine the potential functions and prognostic value of CXCLs. In addition, we verified the immune-related functions of CXCLs in HNSC using single-cell datasets and analyzed the effect of CXCL expression on the survival of HPV-positive and -negative patients.

## RESULTS

### CXCL transcript expression in HNSC patients

**Figure 1:**
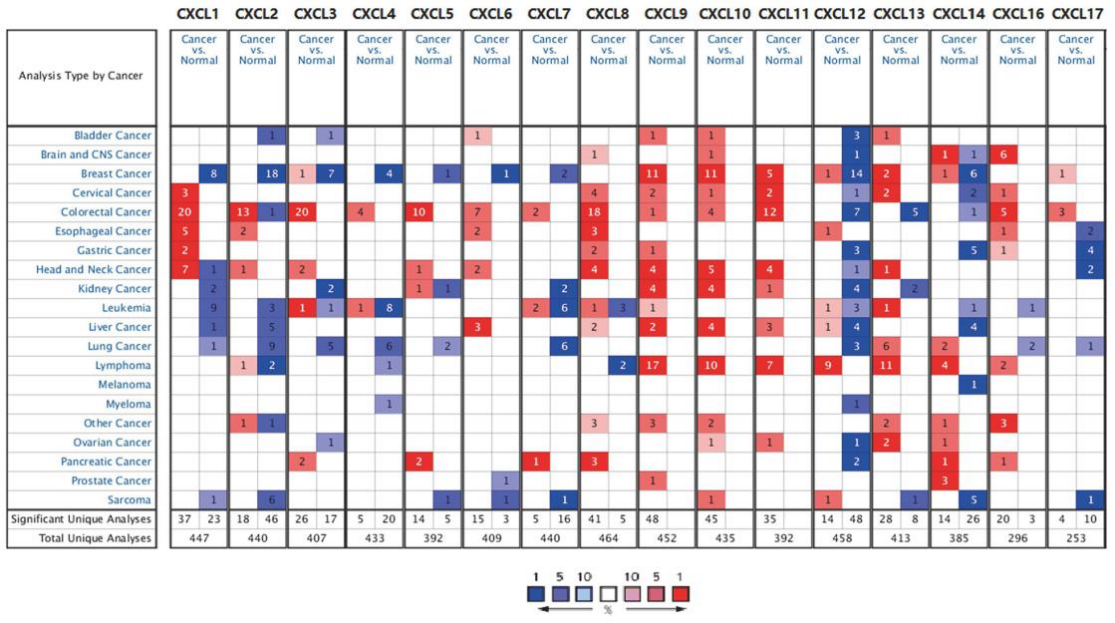
**CXC chemokine mRNA expression in different types of cancer.** Number of datasets with statistically significant CXC chemokine mRNA expression: red, upregulated; blue, downregulated. Fold changes and *p* values are shown in Supplementary Table 2.

**Figure 2:**
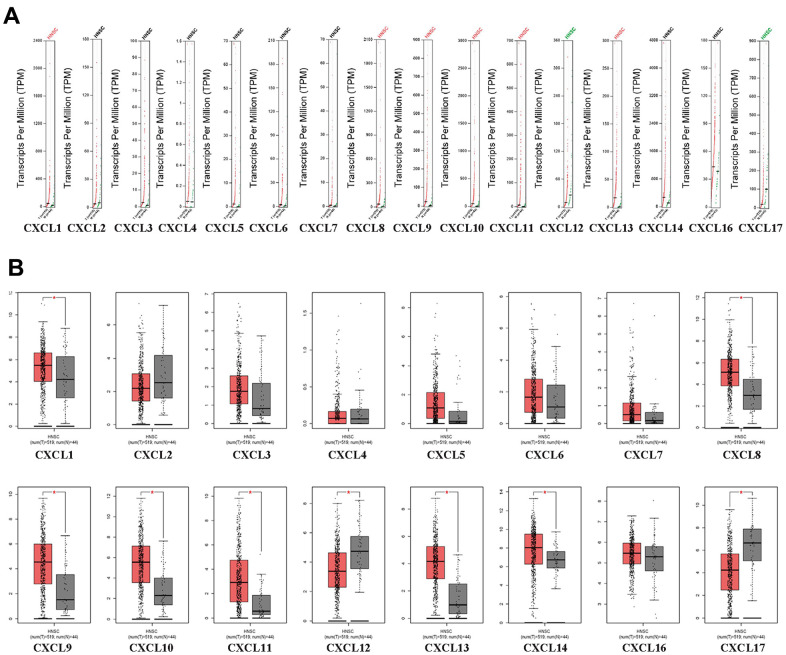
**CXC-motif chemokine ligand (CXCL) expression in head and neck squamous cell carcinoma (HNSC).** (**A**) Differential scatter diagram of CXCL expression in HNSC: red, upregulated; green, downregulated. (**B**) Differential box plot of CXCL expression in HNSC. *p < 0.05.

First, we used Oncomine to compare the expression of the 16 CXCLs identified in human cells between HNSC and normal samples (Figure 1). Although the mRNA expression of CXCL12 and 17 was decreased in two datasets, that of all other CXCLs was significantly upregulated in HNSC patients from seven datasets [29–35] (Supplementary Table 2). Therefore, we compared the mRNA expression of CXCLs between HNSC and normal tissues using GEPIA. The expression of CXCL1, 8–11, and 13 was higher, while that of CXCL12 and 17 was lower, in HNSC than in normal tissues (Figure 2).

To verify the differences in CXCL expression between HNSC tumor and normal tissues as well as between HPV-positive and -negative HNSC patients, we used the Tumor IMmune Estimation Resource (TIMER) database. Consistently, various CXCLs were expressed differently among these samples, with CXCL5, 7–10, 13, 14, 16, and 17 displaying clear differences between HPV-positive and -negative patients (Supplementary Figures 1, 2). In addition, only CXCL9–12 and 14 expression varied significantly with HNSC tumor stage (Supplementary Figure 3). Together, these differences in the expression of members of the CXCL family in HNSC indicate that CXCLs play different but important roles in the HNSC tumor microenvironment.

### CXCL gene expression and mutation in HNSC patients

**Figure 3:**
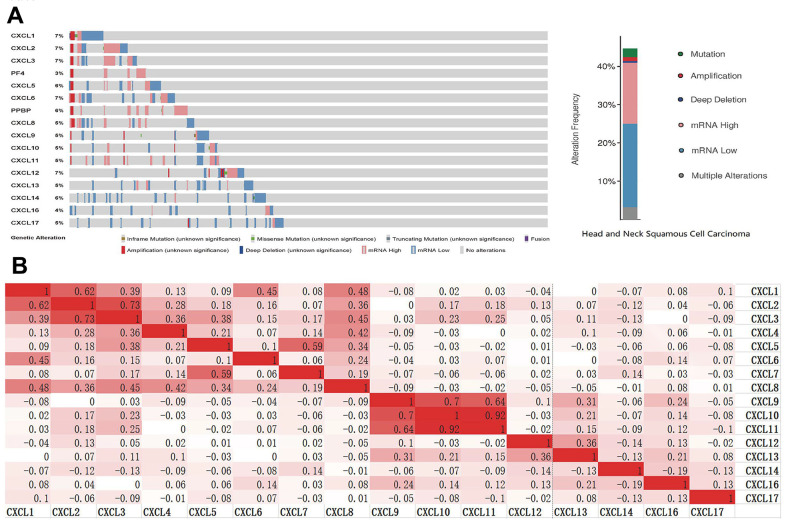
**CXC-motif chemokine ligand (CXCL) gene expression and mutation analysis in head and neck squamous cell carcinoma (HNSC).** (**A**) CXCL gene expression and mutations in HNSC were analyzed using cBioPortal. (**B**) The associations among CXCLs in HNSC were analyzed using the cBioPortal database. Darker colors indicate a stronger correlation.

To analyze the changes in CXCL expression and their correlations with HNSC, we used the cBioPortal online tool. CXCL expression was altered in samples collected from 488 HNSC patients, whereas the queried genes were altered in 218 (45%) of the 488 queried patients (Figure 3A). We also used cBioPortal to analyze CXCL mRNA expression (log RNA sequencing [RNA-Seq] version (v.)2 RSEM) and the correlations among CXCLs using Pearson’s correction. The following significant positive correlations were identified: CXCL1 correlated with CXCL2, 3, 6, and 8; CXCL2 with CXCL1, 3, and 8; CXCL3 with CXCL1, 2, 4, 5, and 8; CXCL4 with CXCL3 and 8; CXCL5 with CXCL3, 7, and 8; CXCL6 with CXCL1; CXCL7 with CXCL5; CXCL8 with CXCL1, 2, 3, 4, and 5; CXCL9 with CXCL10, 11, and 13; CXCL10 with CXCL9 and 11; CXCL11 with CXCL9 and 10; CXCL12 with CXCL13; and CXCL13 with CXCL9 and 12 (Figure 3B). These relationships were verified using the TIMER database, with similar outcomes (Supplementary Figure 4), suggesting that the functions of multiple members of the CXCL family in HNSC are related.

### Predicted functions and pathways of CXCL-related genes in HNSC patients

**Figure 4:**
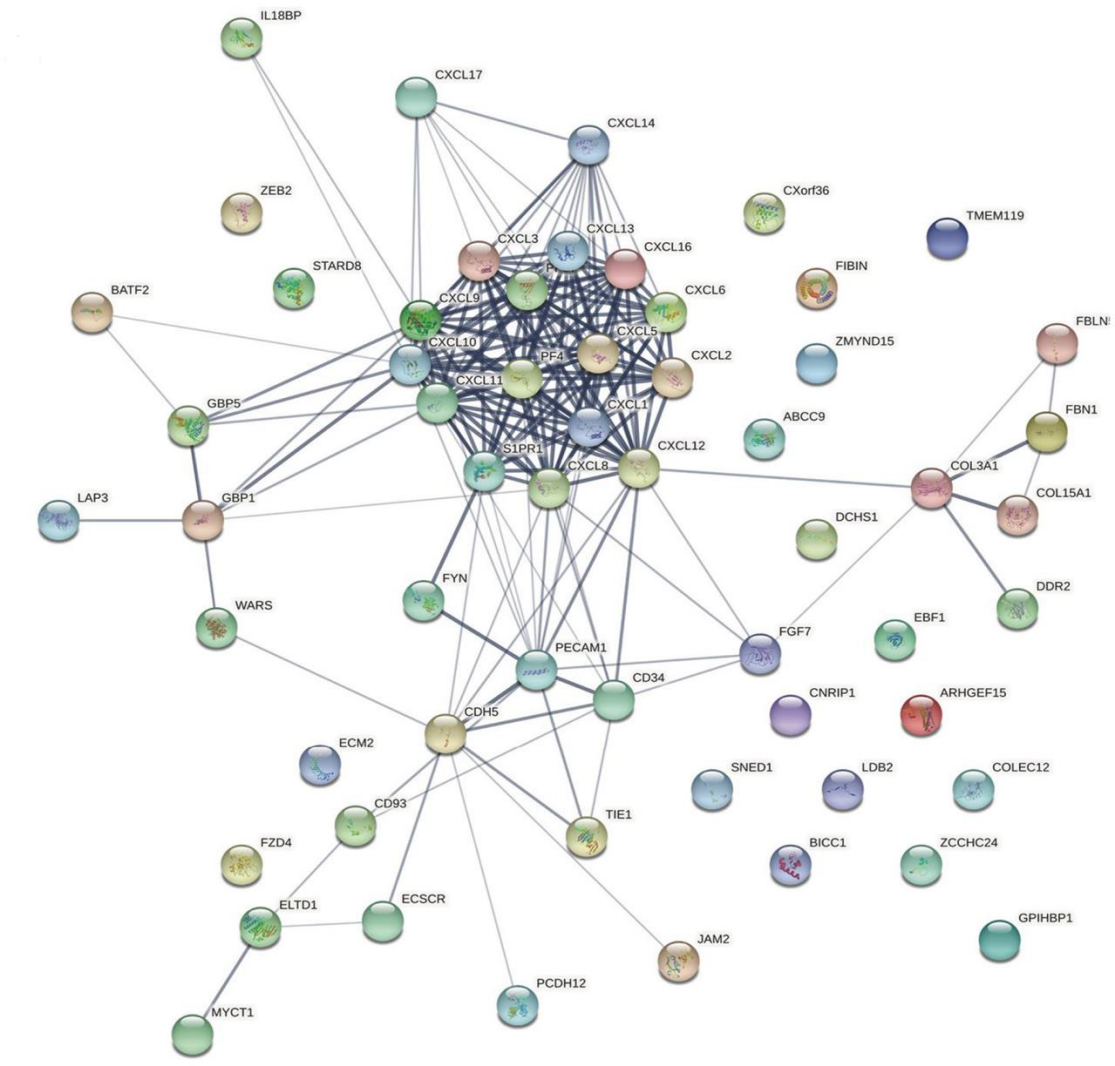
**Protein–protein interaction network.** The relationships between CXCLs and the top 50 similar genes were visualized using the STRING database (see Supplementary Table 3 for a detailed gene list).

The top 50 CXCL-related genes were detected using GEPIA2 (Supplementary Table 3) and then used to produce a network with CXCLs using String to visualize the relationships among them (Figure 4). Another network was produced using Metascape to visualize the function of these genes (Supplementary Figure 5), as follows: extracellular structure organization, blood vessel morphogenesis, cell–substrate adhesion, endothelium development, platelet alpha granule, calcium ion binding, calcium-dependent cell–cell adhesion via plasma membrane cell adhesion molecules, cytokine binding, protein homodimerization activity, protein localization to cell surface, regulation of bone mineralization, extracellular matrix glycoproteins, peptidyl-tyrosine phosphorylation, collagen trimer, cellular response to transforming growth factor beta stimulus, response to wounding, G protein-coupled receptor binding, cell surface interactions at the vascular wall, Rho GTPase cycle, and camera-type eye development.

**Figure 5:**
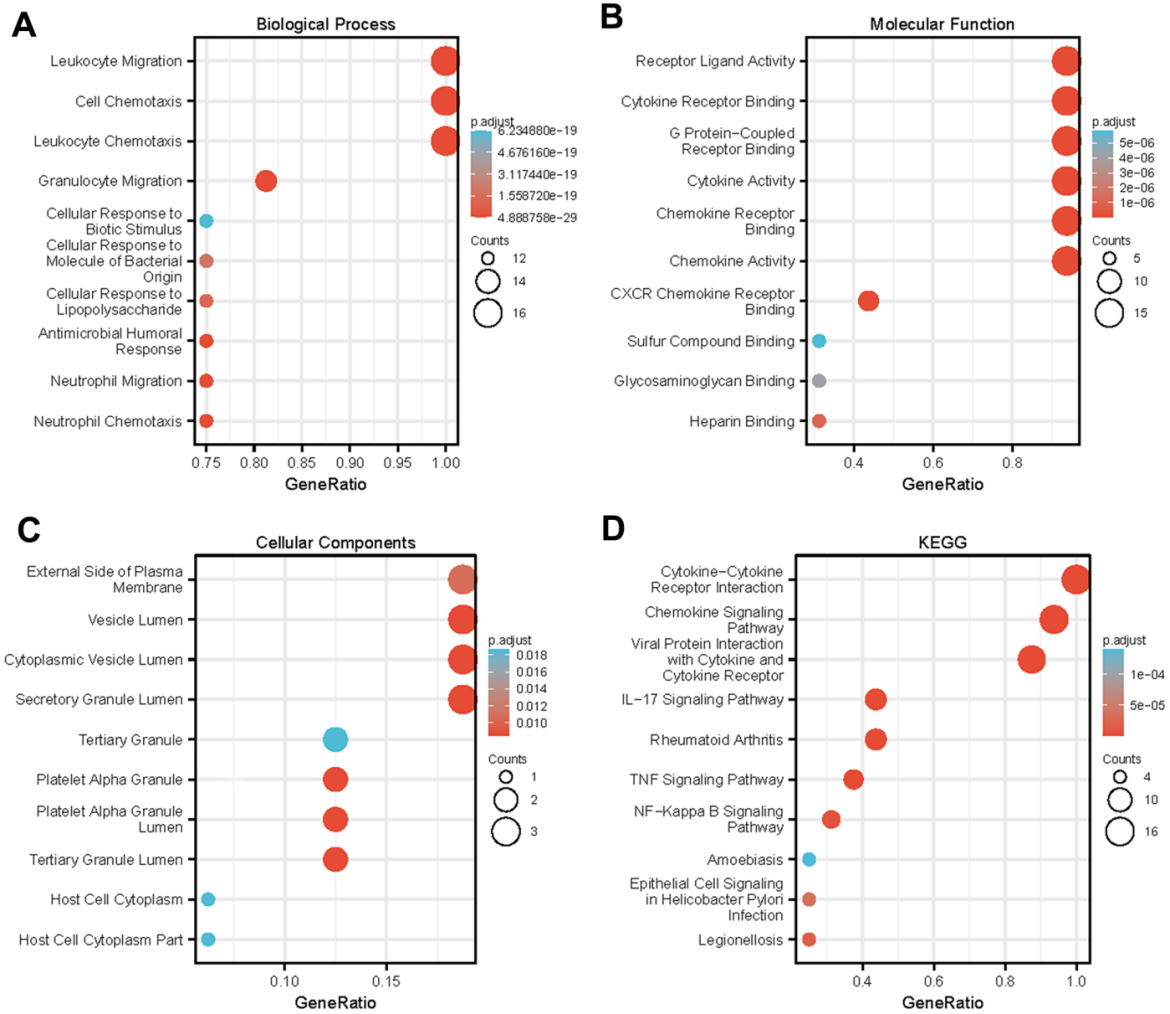
**Enrichment analysis of differentially expressed CXC-motif chemokine ligands (CXCLs) in head and neck squamous cell carcinoma (HNSC).** GO enrichment analysis predicted the functional roles of target genes based on three aspects, including (**A**) biological processes (BP), (**B**) molecular functions (MF), and (**C**) cellular components (CC). (**D**) Kyoto Encyclopedia of Genes and Genomes (KEGG) analysis.

**Figure 6:**
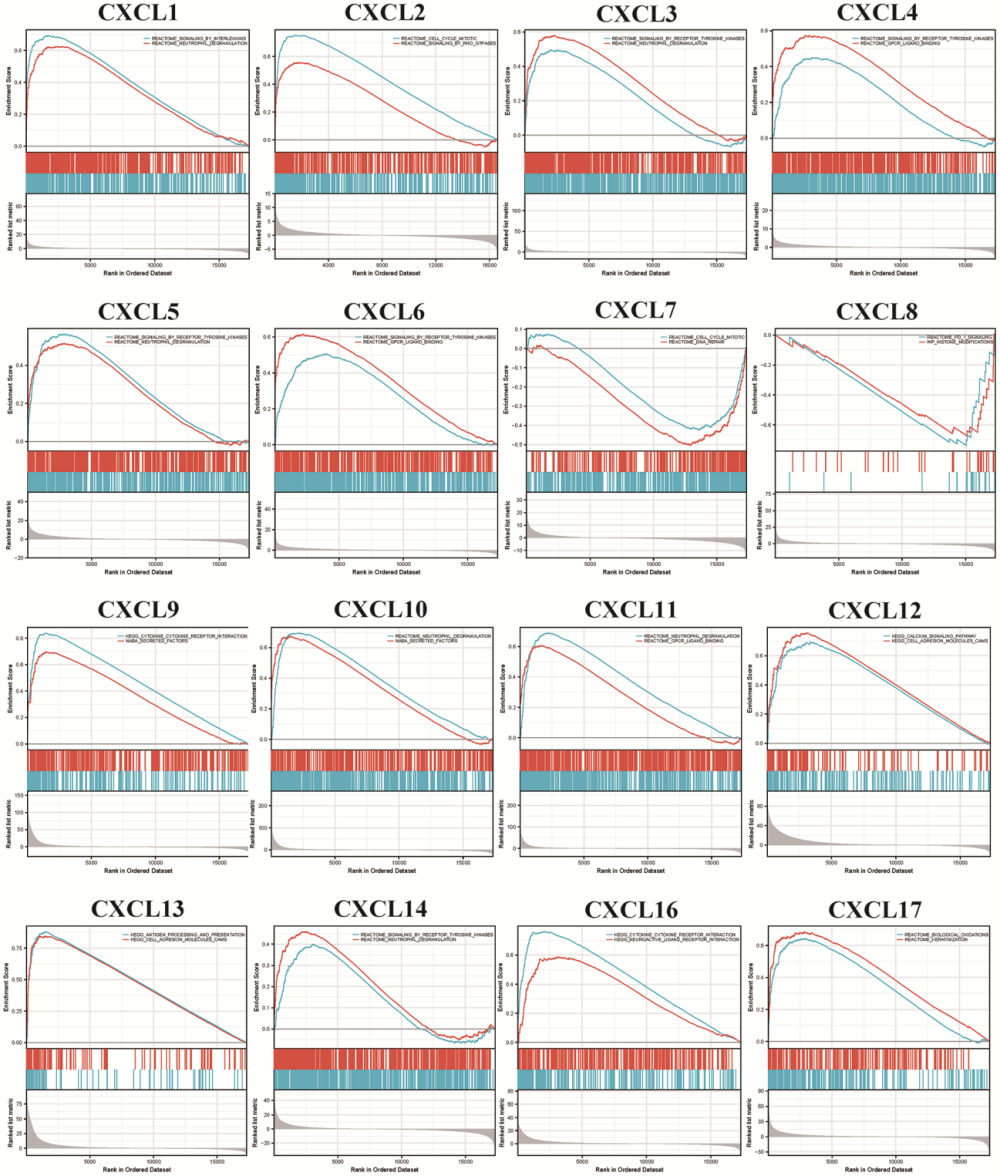
Gene set enrichment analysis (GSEA) analyzed the Kyoto encyclopedia of genes and genomes (KEGG) enrichment of CXC-motif chemokine ligands (CXCLs) in head and neck squamous cell carcinoma (HNSC).

The functions of the CXCLs were predicted using Gene Ontology (GO) analysis and Kyoto Encyclopedia of Genes and Genomes analysis (Figure 5) and visualized using the R package clusterProfiler [36]. In order to get more accurate results, we next performed Gene Set Enrichment Analysis (GSEA). We used the RNAseq datatype of TCGA-HNSC in the Linkedomics database. We set the minimum number of genes as 3 and the simulations were 500. Results were visualized using ggplot2 Rpackage (Figure 6). Taken together, the results of these functional enrichment analyses suggest that CXCLs play an important role in the HNSC tumor microenvironment via immune-related signaling pathways, which have been unreported previously.

### CXCL expression in different immune cells in HNSC

**Figure 7:**
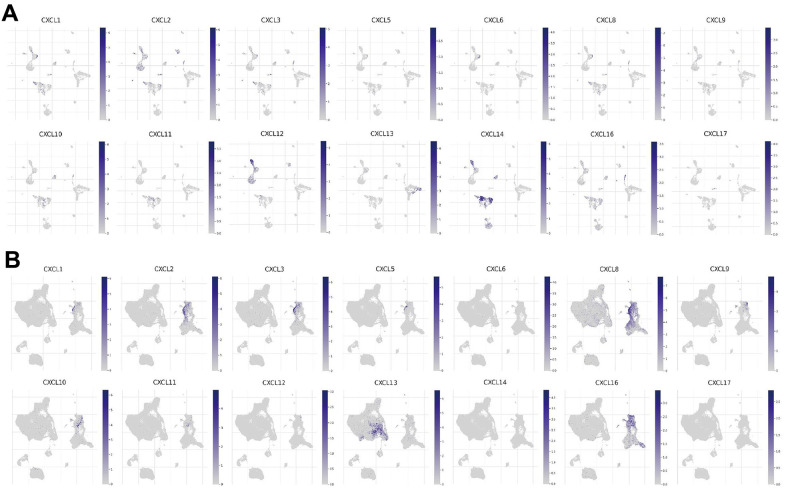
**CXC-motif chemokine ligand (CXCL) expression in two single-cell datasets.** CXCL expression in different immune cells in (**A**) HNSC_GSE103322 and (**B**) HNSC_GSE139324.

**Figure 8:**
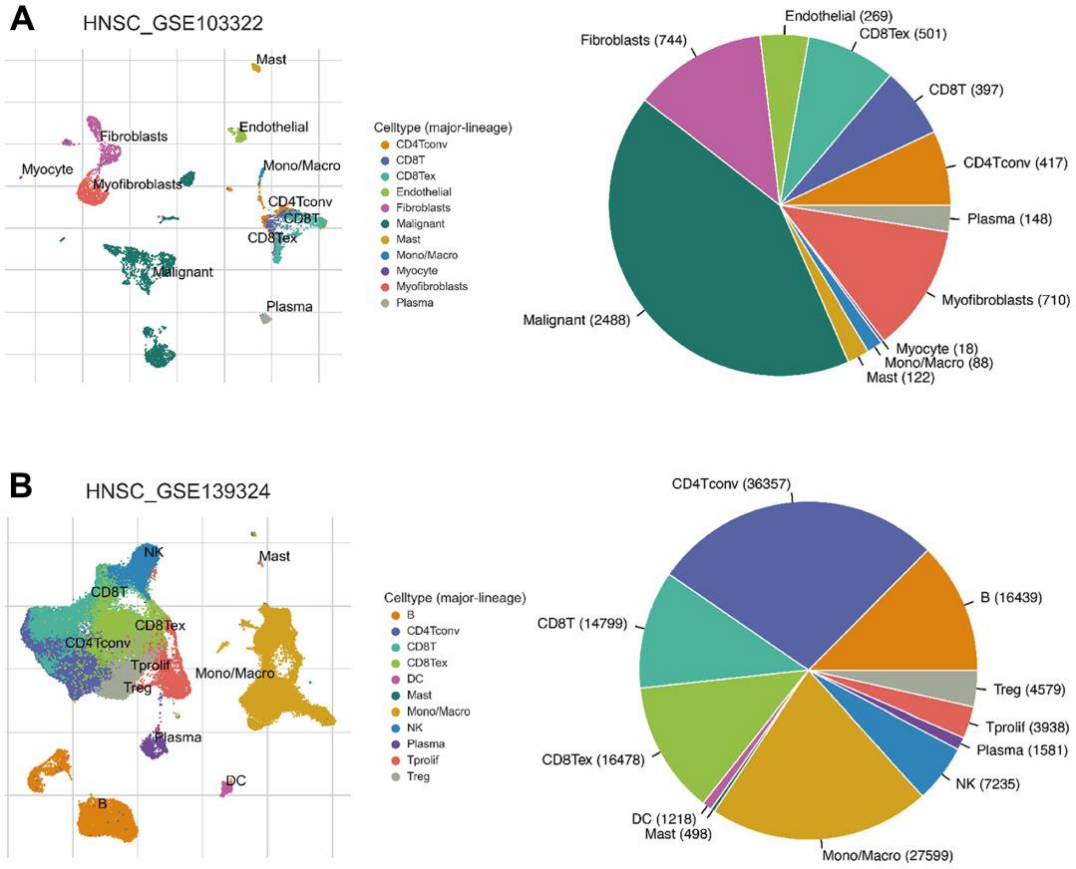
**Infiltration site distribution (left) and proportion (right) of different cell types in HNSC.** (**A**) HNSC_GSE103322. (**B**) HNSC_GSE139324.

Since the enriched pathways were related to immune function, we analyzed CXCL expression in two datasets from the Tumor Immune Single-cell Hub (TISCH) database: HNSC_GSE103322 (5,902 cells) and HNSC_GSE139324 (130,721 cells) (Figure 7). Detailed information about the distribution and proportion of immune cells in each dataset is shown in Figure 8. We found that CXCL2, 3, 8, 10, 12–14, and 16 expression differed in immune cells of the HNSC_GSE103322 dataset (Supplementary Table 4), whereas CXCL1–3, 8–10, 13, and 16 expression differed in the immune cells of HNSC_GSE139324 (Supplementary Table 5). This differential expression of CXCL1–3, 8–10, 12–14, and 16 in different immune cell types in HNSC suggests that they play an important role in the tumor microenvironment by affecting immune cells.

### CXCLs and immune cell infiltration in HNSC patients

Since TISCH analysis revealed that CXCLs are involved in inflammatory responses and immune cell infiltration in HNSC patients, we comprehensively explored the correlations between differentially expressed CXCLs and immune cell infiltration using the TIMER database (Supplementary Figure 6). Notably, CXCL2, 3, 5, 7–14, 16, and 17 were related to various types of immune cells (Supplementary Table 6). Therefore, these findings suggest that CXCL2, 3, 8, 10, 13, and 16 greatly affect immune functions and thereby influence HNSC tumorigenesis and tumor development.

### Association between CXCL mRNA expression and HNSC prognosis

**Figure 9:**
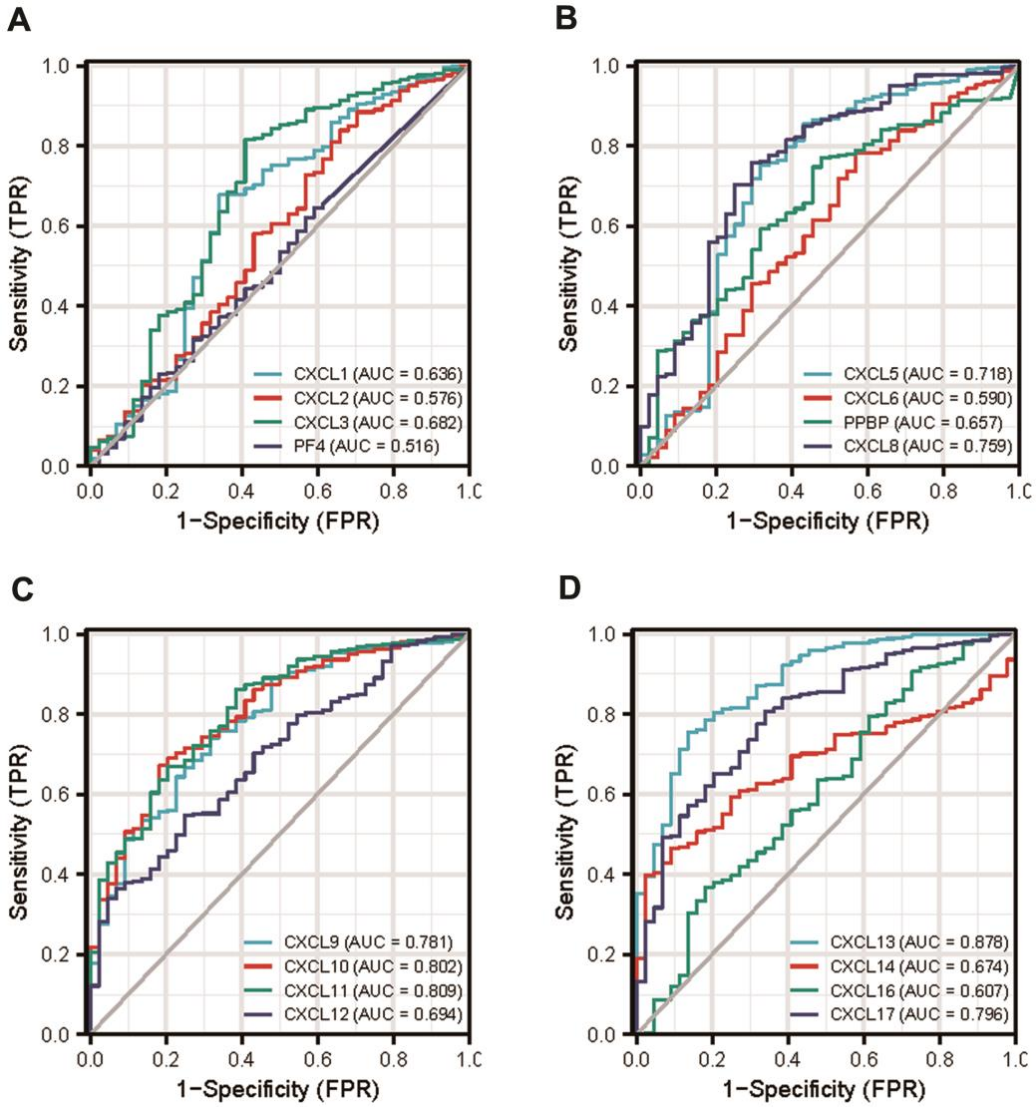
**Diagnostic efficacy of CXC-motif chemokine ligands (CXCLs) in head and neck squamous cell carcinoma (HNSC).** (**A**) Receiver operating characteristic curve (ROC) of CXCL1, 2, 3, 4. (**B**) ROC of CXCL5, 6, 7, 8. (**C**) ROC of CXCL9, 10, 11, 12. (**D**) ROC of CXCL13, 14, 16, 17. TPR: True Positive Rate, FPR: False Positive Rate.

Next, we used the data from The Cancer Genome Atlas (TCGA) to obtain receiver operating characteristic curve (ROC) to evaluate the diagnostic efficacy of CXCLs in the development of HNSC (Figure 9). The area under the curve value of CXCL10, 11, and 13 was 0.8 fold higher than that of other CXCLs, indicating that the expression of these genes could distinguish tumors from non-tumors and used as a potential diagnostic biomarker.

**Figure 10:**
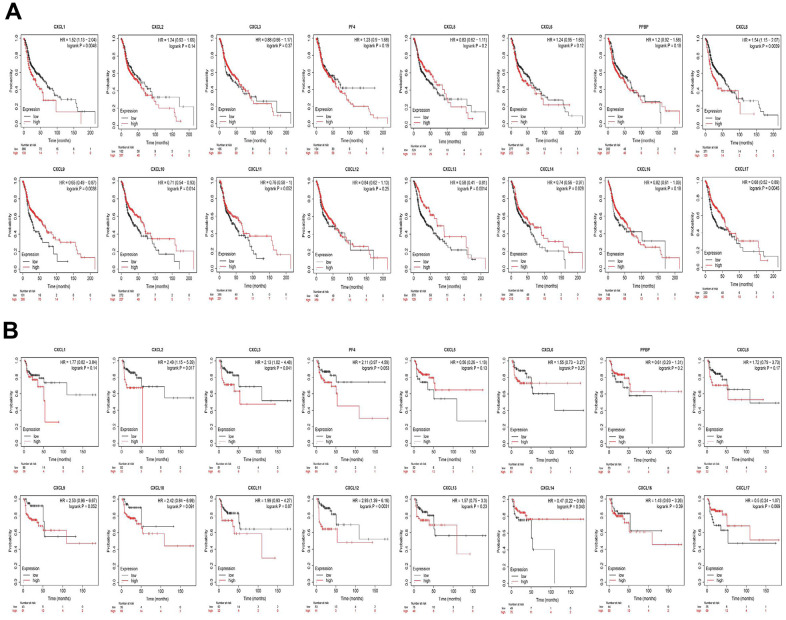
**Prognostic value of CXC-motif chemokine ligands (CXCLs) in head and neck squamous cell carcinoma (HNSC).** (**A**) Prognostic value of CXCLs for the overall survival of HNSC patients. (**B**) Prognostic value of CXCLs for the recurrence-free survival of HNSC patients.

**Figure 11:**
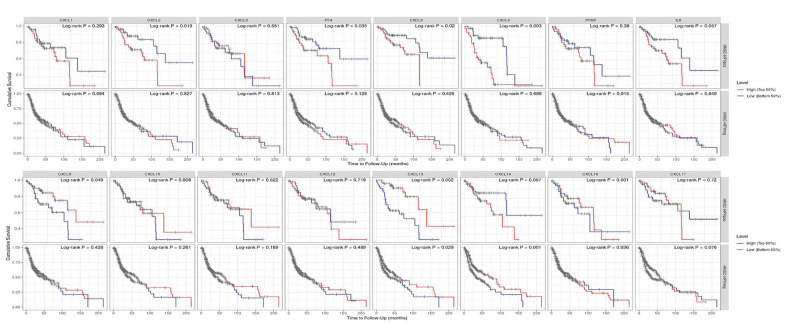
**Prognostic value of CXCLs for the overall survival of HPV-positive and -negative head and neck squamous cell carcinoma (HNSC) patients.** Upper: HNSC-HPVpos, HPV-positive HNSC patients. Lower: HNSC-HPVneg, HPV-negative HNSC patients.

Finally, we explored the effect of CXCL mRNA expression on the survival of HNSC patients using Kaplan–Meier Plotter curve and log rank test analyses. Decreased CXCL1, 2, 4, and 6–8 mRNA levels and increased CXCL9–14 and 17 mRNA levels were significantly associated with the overall survival (OS) of all HNSC patients (Figure 10A). In addition, patients with high CXCL5, 7, 14, or 17 mRNA levels or low CXCL1–4, 8, 11, or 12 mRNA levels were predicted to have high recurrence-free survival (RFS; Figure 10B). Therefore, we used TIMER to analyze the relationship between immune cell number and cumulative survival (Supplementary Figure 7), as well as the relationship between CXCL expression and the cumulative survival of HPV-positive and -negative HNSC patients. Decreased CXCL1, 2, 4–8, and 14 expression and increased CXCL9 and 13 expression were associated with high survival in HPV-positive HNSC patients; however, only high expression levels of CXCL13 and 14 were related to improved prognosis in HPV-negative HNSC patients (Figure 11). Together, our analysis of the relationship between CXCL expression and the prognosis of HNSC patients in large scale data from different databases verified that CXCL family members could exert significantly different effects on the long-term survival of HNSC patients.

## DISCUSSION

CXCLs play key roles in many cancers and are associated with diverse regulatory functions in HNSC [37]. Although some studies have reported the roles of various CXCLs in HNSC [38–40], no systematic analyses have been performed on this gene family. Moreover, few studies have investigated the relationship between CXCLs and the tumor microenvironment in HNSC. To our knowledge, this study is the first to analyze single-cell databases to explore the immune roles of CXCLs in the HNSC tumor microenvironment and identify key CXCLs that may play important roles in tumorigenesis and tumor progression.

Initially, we analyzed the relationship between CXCLs and HNSC stage and found that CXCL9–12 and 14 were differentially expressed in different tumor stages. Notably, CXCL9–11 and 14 were highly expressed in HNSC in the Oncomine, GEPIA, and TIMER databases and were associated with good prognosis. Therefore, changes in the expression of these molecules are likely associated with tumor progression and should be detected during the diagnosis, treatment, and prognostic evaluation of HNSC patients. Consistently, Yang et al*.* [39] reported that CXCL1 correlated negatively with the 5-year OS of oral squamous cell carcinoma patients (*p* < 0.05), whereas CXCL10 and 9 correlated positively with their 5-year OS (*p* < 0.05). Moreover, we found that high CXCL8 expression was associated with poor prognosis in HNSC, whereas high CXCL13, 14, and 17 expression may improve the OS of these patients; however, we found no difference between the expression of these molecules and RFS. Low CXCL2, 3, and 12 mRNA levels predicted high RFS, suggesting that they play important regulatory roles in promoting tumor recurrence. Although few studies have investigated CXCL14, high CXCL14 expression has been found to suppress tumor growth in HNSC [41]. We found that CXCL14 is associated with tumor stage, OS, and RFS, suggesting that it could be an important target for treating HNSC.

Previously, Szabo et al. [42] demonstrated that the upregulation of CXCL1 and 8 is not cancer specific, supporting the hypothesis that similar mechanisms exist in wound healing and oncogenesis. Here, we found a strong positive correlation between CXCL1 and 8 as well as many other CXCLs. Although most studies have focused on the roles of individual CXCLs in tumors, our results suggest that multiple molecules exert a combined effect on the tumor microenvironment. However, we found that CXCL4 is not associated with HNSC but is closely related to periodontitis and significantly and positively correlated with CXCL3 and 8. An in-depth understanding of the tumor microenvironment and tumorigenesis mechanism would clarify whether CXCL4, along with CXCL3 and 8, exerts potential synergistic effects on HNSC tumorigenesis.

In this study, our systematic enrichment analysis of CXCLs identified several signaling pathways that have not been previously reported to be involved in the function of CXCLs in HNSC. These findings suggest that HNSC progression is closely related to CXCL-mediated inflammation. Further analysis of single-cell datasets showed that the expression of many CXCLs was closely related to the role of immune cells in HNSC, confirming our hypothesis; however, CXCL16 was not closely associated with HNSC. Although no studies have yet reported the correlation between CXCL16 and HNSC, we observed a significant difference in CXCL16 expression in classically (M1) and alternatively (M2) activated macrophages in HNSC, suggesting that CXCL16 has immunomodulatory roles in HNSC. Further studies with more clinical samples are required to elucidate the specific molecules and mechanisms involved.

In conclusion, this study provides a thorough understanding of the heterogeneity and complexity of the molecular biological properties of HNSC by analyzing the expression and prognostic value of CXCLs in HNSC. In addition, we verified the function of CXCLs as biomarkers in HNSC and found, for the first time, that CXCL16 may play a significant role in the occurrence and development of HNSC by modulating immunity. Furthermore, we revealed that CXCL13 and 14 may be exclusive biomarkers for HPV-negative HNSC and that the top 50 CXCL-related genes enriched for signaling pathways may be closely related to HNSC development. Because of the lack of detailed single-cell data for HNSC, we were unable to analyze the role of CXCLs in immune infiltration in greater detail; therefore, we aim to improve these data in the future and conduct in-depth analyses to identify more significant and meaningful biomarkers, particularly for HPV-negative HNSC. Taken together, we believe that our findings improve the current knowledge of HNSC and could improve the diagnostic accuracy, treatment, and prognosis of HNSC patients.

## MATERIALS AND METHODS

### Oncomine analysis

Oncomine is an online cancer gene expression microarray database that can be used to analyze CXCL transcription levels in different cancers (https://www.oncomine.org/resource/login.html) [43]. In this study, we compared CXCL mRNA expression in clinical cancer tissues with that in normal control tissues, with *p* value and fold change cutoffs of 0.05 and 2, respectively.

### GEPIA dataset

GEPIA (http://gepia.cancer-pku.cn/) is a newly developed interactive web server that can analyze the RNA sequencing expression data of 9,736 tumors and 8,587 normal samples from TCGA and the Genotype Tissue Expression (GTEx) project using a standard processing pipeline [44]. Using this database, we verified differences in CXCL expression between tumor and normal tissues and identified correlations among CXCLs. The top 50 CXCL-related genes were detected using GEPIA2, a python package that provides rapid analysis and result retrieval.

### cBioPortal

The cBio Cancer Genomics Portal (cBioPortal, http://www.cbioportal.org/) provides data for more than 5,000 tumor samples from 20 cancer studies [45, 46]. The genomic profiles (523 samples) include mutations, putative copy number alterations from the genomic identification of significant targets in cancer, and mRNA expression z scores relative to all samples (log RNA Seq V2 RSEM) with a threshold of ±2.0.

### String

To visualize the relationships among the top 50 CXCL-related genes, we used the String database (https://string-db.org/), which uses a spring model to generate network images in which nodes are modeled as masses and edges are modeled as springs. Final node position is calculated by minimizing the “energy” of the system. High confidence edges are given a higher “spring strength” so that they reach the best position before lower confidence edges. By default, the database sets high confidence edge length to 80% of the normal length [47–57].

### Metascape

Metascape (http://metascape.org/) is a web-based portal that allows experimental biologists to analyze and annotate using a comprehensive gene list. In particular, Metascape combines functional enrichment, interactive group analysis, gene annotation, and member search by combining more than 40 independent knowledge bases in one integrated portal. In addition, Metascape can conveniently compare and analyze datasets from several independent and orthogonal experiments [58]. In this study, we used Metascape to visualize the GO enrichment results.

### TISCH

The TISCH (http://tisch.comp-genomics.org/) is a scRNA-seq database that provides detailed cell-type annotation at the single-cell level to explore the tumor microenvironment across different cancer types. TISCH integrates the single-cell transcriptomic profiles of nearly 2 million cells from 76 high-quality tumor datasets across 27 cancer types [59]. We analyzed CXCL expression in various immune cells at the single-cell level using two HNSC datasets in TISCH.

### Kaplan–Meier plotter

The Kaplan–Meier plotter can assess the effect of 54,000 genes (mRNA, miRNA, and protein) on survival in 21 cancer types using source databases such as the intergovernmental Group on Earth Observations, European Genome-phenome Archive, and TCGA [60]. We analyzed OS and RFS using “autoselect best cutoff,” wherein all possible cutoff values between the lower and upper quartiles were computed, and the best performing threshold was used as a cutoff. Data from patients that survived over the selected follow-up threshold were not used to generate the plot.

### TIMER

The TIMER database is a network resource that systematically evaluates the clinical effects of different immune cells in different types of cancer (http://cistrome.dfci.harvard.edu/TIMER/). A new statistical method produced by the developers was used to estimate the abundance of six immune cell types in the tumor microenvironment: B cells, CD4 T cells, CD8 T cells, neutrophils, macrophages, and dendritic cells [61–63]. We used TIMER to analyze the correlation among CXCLs and between CXCL expression and the infiltration of various immune cell types. In addition, we analyzed the difference in CXCL expression between tumor and normal tissues as well as between HPV-positive and -negative tumor tissues and analyzed the effect of CXCLs on OS in HPV-positive and -negative HNSC.

### Data availability

The differences in gene expression were analyzed using Oncomine, GEPIA, TIMER, and TCGA (https://portal.gdc.cancer.gov/), while the relationships among CXCLs were identified using cBioPortal. The top 50 most similar genes were obtained using GEPIA, and the resulting network was assembled and analyzed using String, Metascape, LinkedOmics [64], and DAVID. We confirmed our results using two single-cell datasets (HNSC_GSE103322 and HNSC_GSE139324) from TISCH and analyzed immune infiltration using TIMER. Finally, the survival curves were analyzed using the Kaplan–Meier Plotter and TIMER.

### Statistical analysis

All statistical analyses were carried out using SPSS Statistics 20.0 software (IBM SPSS, Chicago, USA). The statistical differences between the experimental groups were analyzed by Student’s *t* test. The relationship between CXCL expression and survival rates was analyzed using Kaplan–Meier (Km) curve. The *p* values were obtained using Student’s *t* test.

## Supplementary Material

Supplementary Figures

Supplementary Tables
