# Supplementary material for: Bioinformatics analysis of the role of CXC ligands in the microenvironment of head and neck tumor
Source: Aging (Albany NY). 2021 Jul 11;13(13):17789–817. doi: 10.18632/aging.203269 (PMC8312447; doi:10.18632/aging.203269)
Supplement: Supplementary Figures [file aging-13-203269-s001.pdf]

SUPPLEMENTARY FIGURES

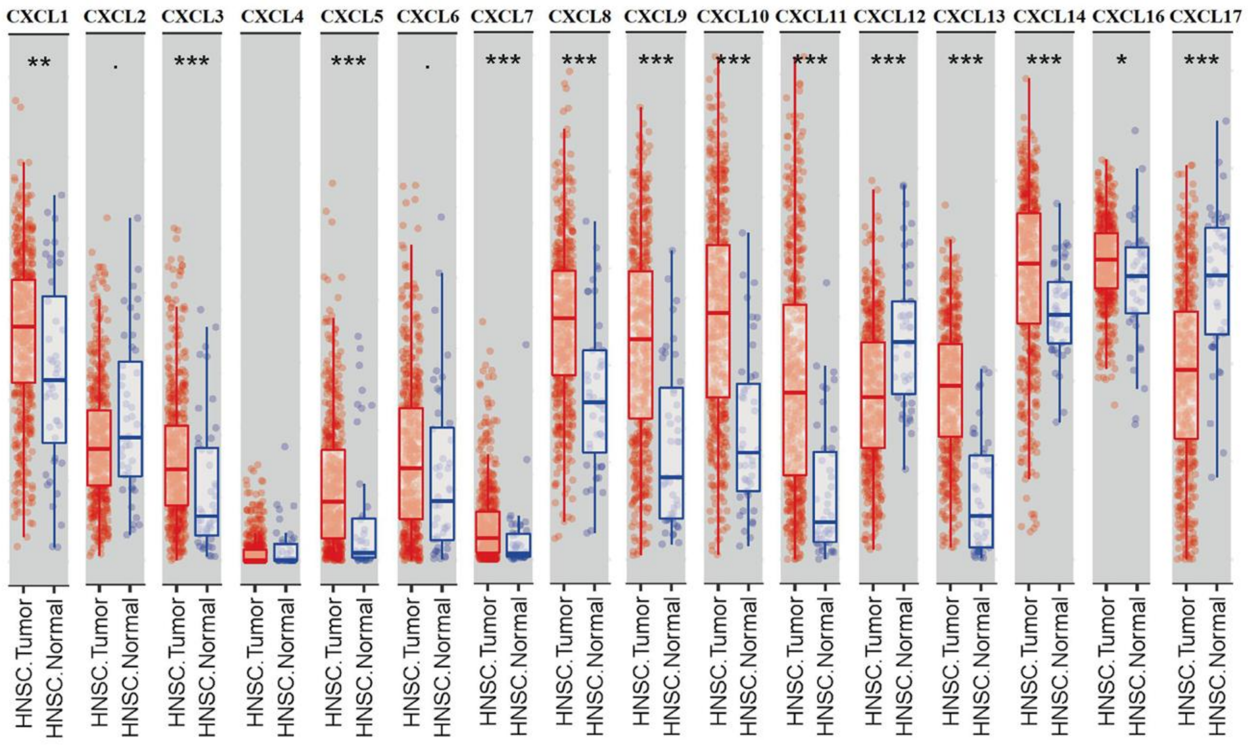

Supplementary Figure 1. CXC-motif chemokine ligand (CXCL) expression in head and neck squamous cell carcinoma (HNSC). Differentially expressed CXCLs in HNSC and normal tissues. *$p < 0.05$; **$p < 0.01$; ***$p < 0.001$.

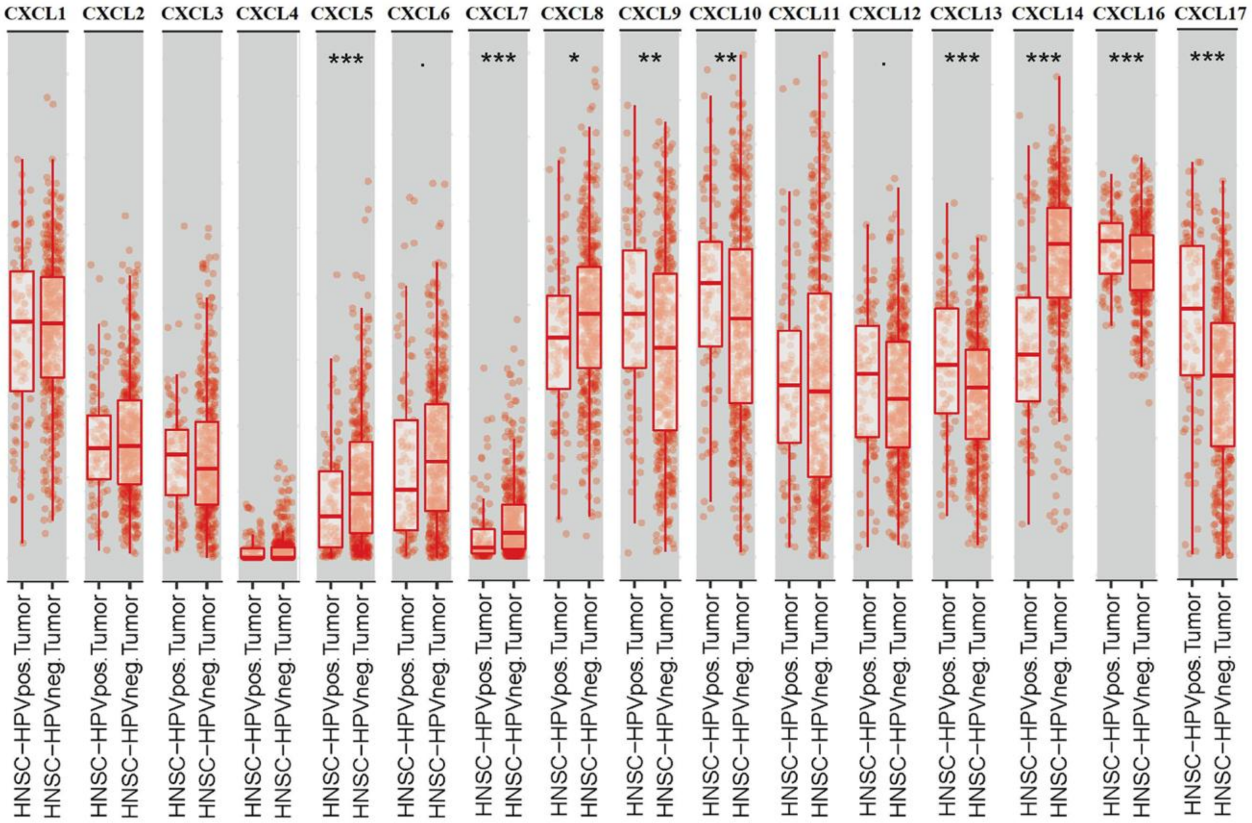

Supplementary Figure 2. CXC-motif chemokine ligand (CXCL) expression in HPV-positive and -negative head and neck squamous cell carcinoma (HNSC) patients. CXCL expression in HPV-positive and -negative HNSC tissues analyzed using the TIMER database. *p < 0.05; **p < 0.01; ***p < 0.001.

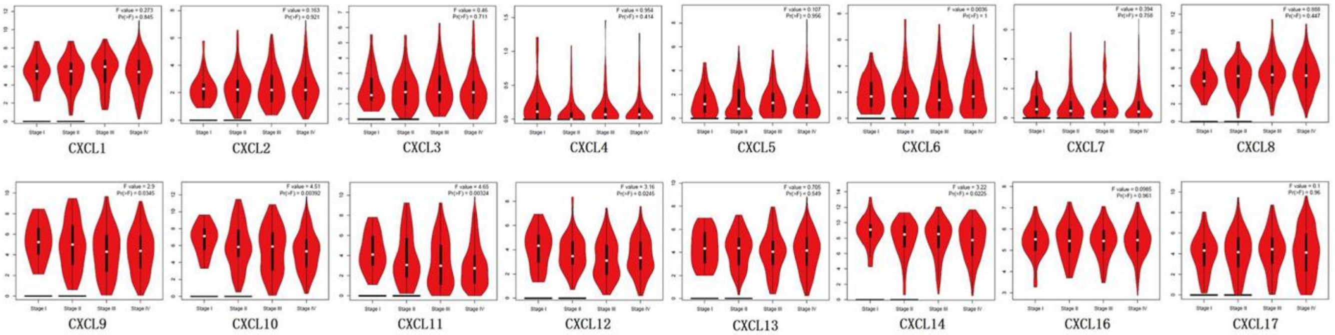

Supplementary Figure 3. CXC-motif chemokine ligand (CXCL) expression and tumor stage in head and neck squamous cell carcinoma (HNSC) patients. CXCL expression at different stages of HNSC was analyzed using the GEPIA database.

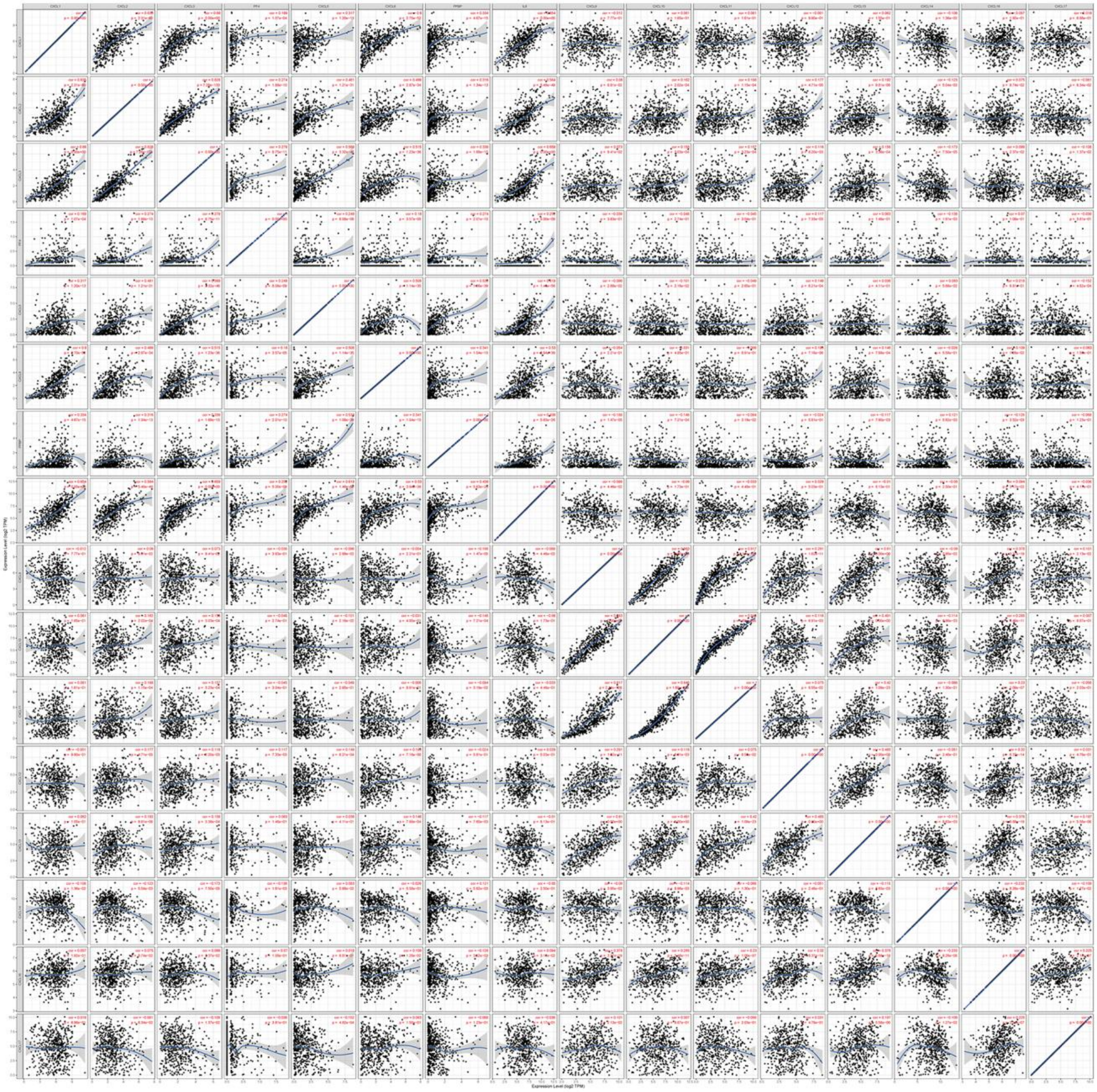

Supplementary Figure 4. Correlation between CXC-motif chemokine ligands (CXCLs) and immune infiltration in head and neck squamous cell carcinoma (HNSC). Immune infiltration was analyzed using the TIMER database to verify the correlation among CXCLs in HNSC.

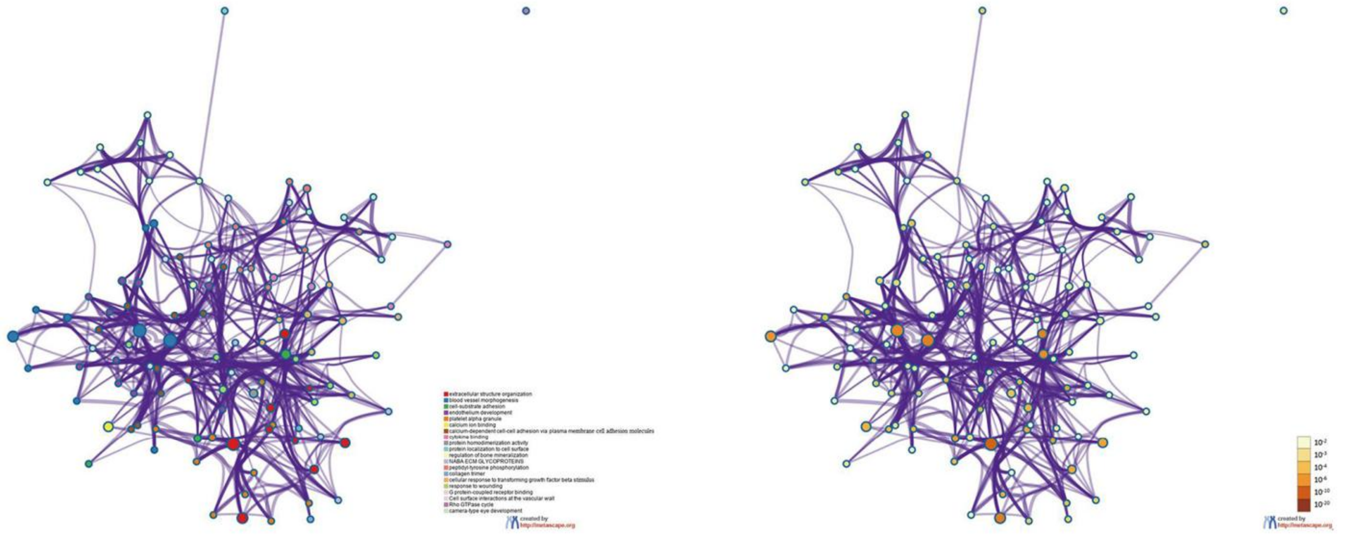

Supplementary Figure 5. Enrichment analysis of differentially expressed CXC-motif chemokine ligands (CXCLs) and the top 50 similar genes in head and neck squamous cell carcinoma (HNSC).

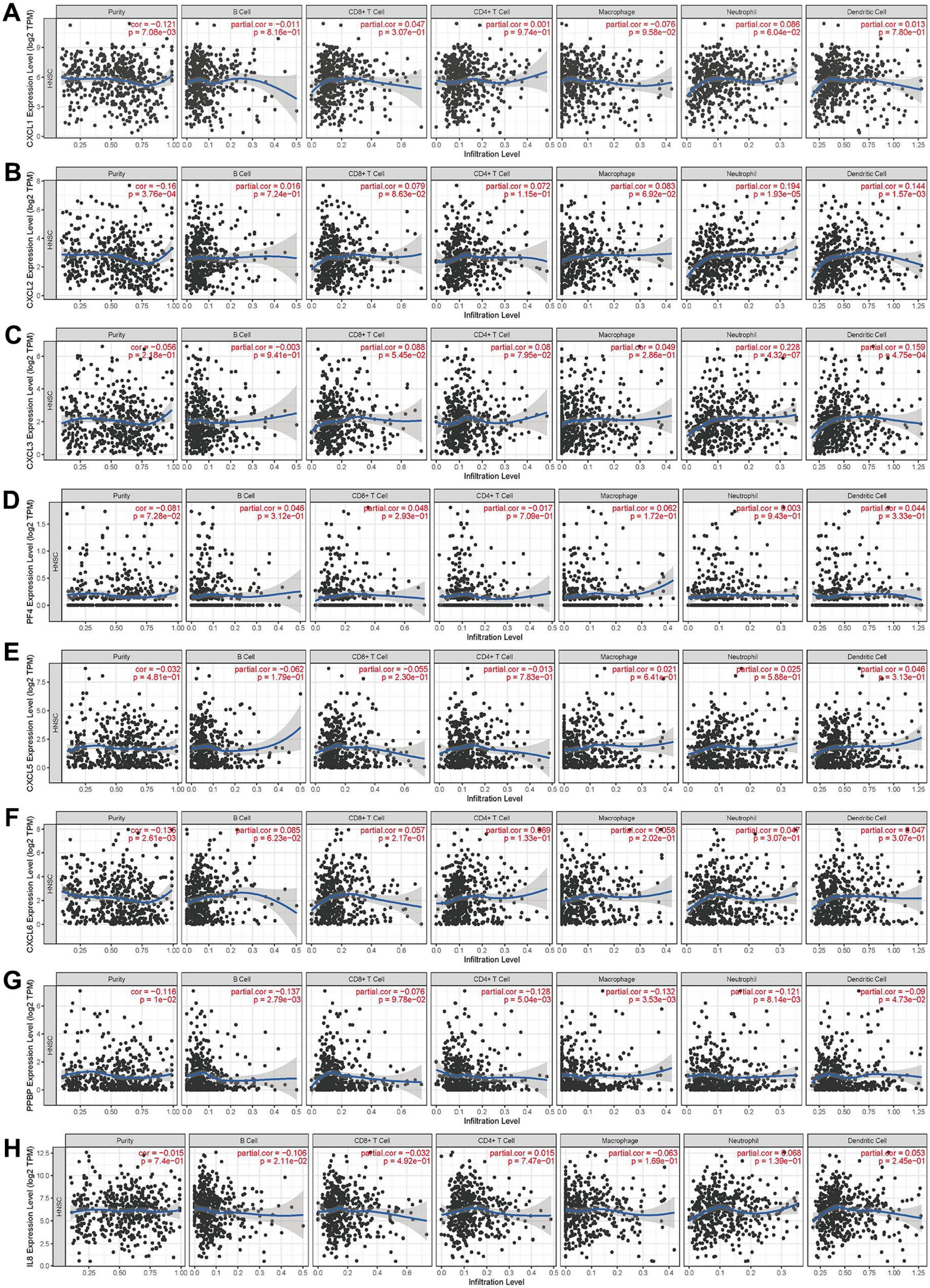
A
B
C
D
E
F
G
H
Purity
B Cell
CD8+ T Cell
CD4+ T Cell
Macrophage
Neutrophil
Dendritic Cell
HNSC
Infiltration Level
CXCL1 Expression Level (log2 TPM)
cor = −0.121 p = 7.08e−03
partial.cor = −0.011 p = 8.16e−01
partial.cor = 0.047 p = 3.07e−01
partial.cor = 0.001 p = 9.74e−01
partial.cor = −0.076 p = 9.58e−02
partial.cor = 0.086 p = 6.04e−02
partial.cor = 0.013 p = 7.80e−01
CXCL2 Expression Level (log2 TPM)
cor = −0.16 p = 3.76e−04
partial.cor = 0.016 p = 7.24e−01
partial.cor = 0.079 p = 8.63e−02
partial.cor = 0.072 p = 1.15e−01
partial.cor = 0.083 p = 6.92e−02
partial.cor = 0.194 p = 1.93e−05
partial.cor = 0.144 p = 1.57e−03
CXCL3 Expression Level (log2 TPM)
cor = −0.056 p = 2.18e−01
partial.cor = −0.003 p = 9.41e−01
partial.cor = 0.088 p = 5.45e−02
partial.cor = 0.08 p = 7.95e−02
partial.cor = 0.049 p = 2.86e−01
partial.cor = 0.228 p = 4.32e−07
partial.cor = 0.159 p = 4.75e−04
PF4 Expression Level (log2 TPM)
cor = −0.081 p = 7.28e−02
partial.cor = 0.046 p = 3.12e−01
partial.cor = 0.048 p = 2.93e−01
partial.cor = −0.017 p = 7.09e−01
partial.cor = 0.062 p = 1.72e−01
partial.cor = 0.003 p = 9.43e−01
partial.cor = 0.044 p = 3.33e−01
CXCL5 Expression Level (log2 TPM)
cor = −0.032 p = 4.81e−01
partial.cor = −0.062 p = 1.79e−01
partial.cor = −0.055 p = 2.30e−01
partial.cor = −0.013 p = 7.83e−01
partial.cor = 0.021 p = 6.41e−01
partial.cor = 0.025 p = 5.88e−01
partial.cor = 0.046 p = 3.13e−01
CXCL6 Expression Level (log2 TPM)
cor = −0.136 p = 2.61e−03
partial.cor = 0.085 p = 6.23e−02
partial.cor = 0.057 p = 2.17e−01
partial.cor = 0.069 p = 1.33e−01
partial.cor = 0.058 p = 2.02e−01
partial.cor = 0.047 p = 3.07e−01
partial.cor = 0.047 p = 3.07e−01
PPBP Expression Level (log2 TPM)
cor = −0.116 p = 1e−02
partial.cor = −0.137 p = 2.79e−03
partial.cor = −0.076 p = 9.78e−02
partial.cor = −0.128 p = 5.04e−03
partial.cor = −0.132 p = 3.53e−03
partial.cor = −0.121 p = 8.14e−03
partial.cor = −0.09 p = 4.73e−02
IL8 Expression Level (log2 TPM)
cor = −0.015 p = 7.4e−01
partial.cor = −0.106 p = 2.11e−02
partial.cor = −0.032 p = 4.92e−01
partial.cor = 0.015 p = 7.47e−01
partial.cor = −0.063 p = 1.69e−01
partial.cor = 0.068 p = 1.39e−01
partial.cor = 0.053 p = 2.45e−01

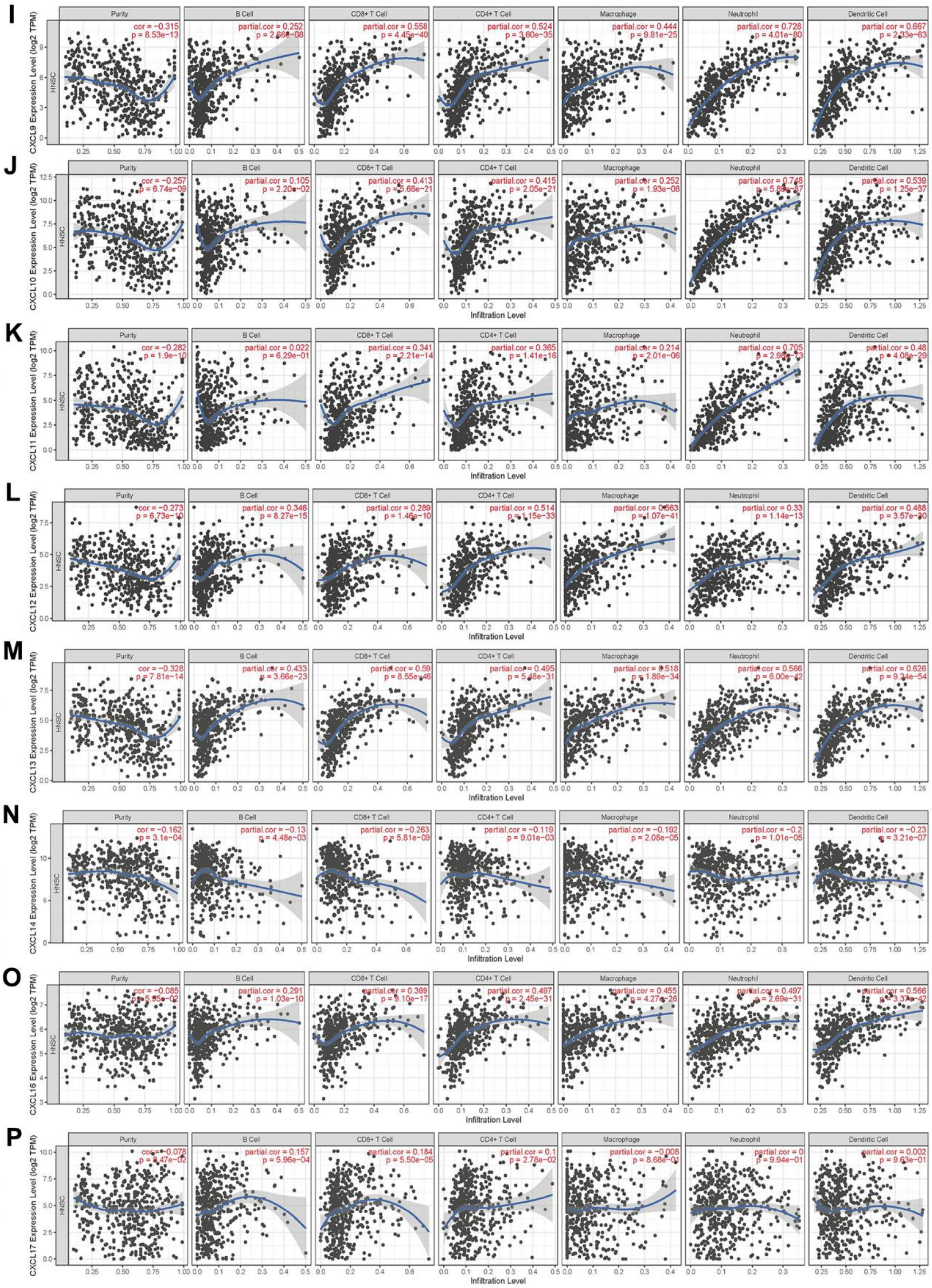

Supplementary Figure 6. Correlation between different expressed CXCLs and immune cell infiltration. (**A**–**P**) Infiltration of CXCL1-14, CXCL16, CXCL17 in B cell, CD8+ T cell, CD4+ T cell, Macrophage, Neutrophil and Dendritic Cell respectively.

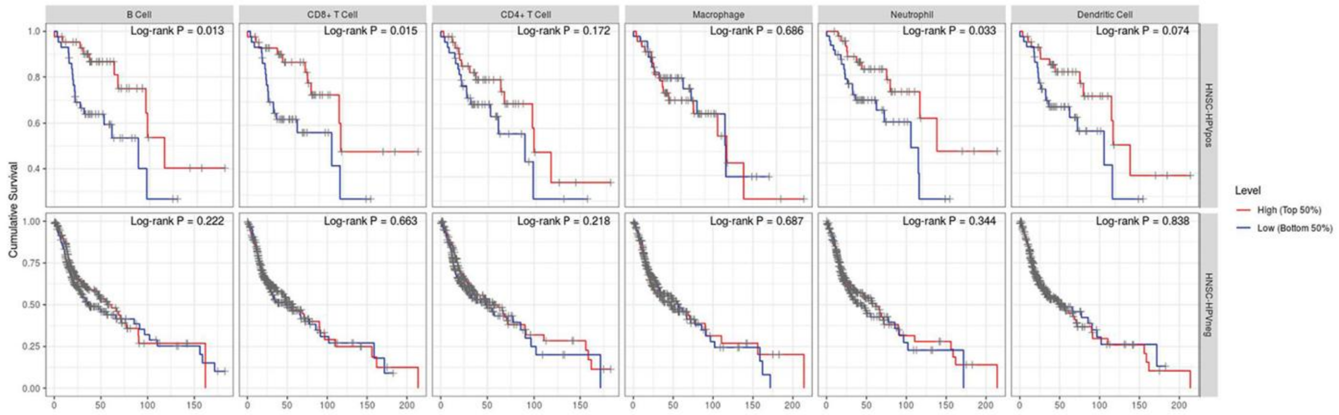

Supplementary Figure 7. Effect of immune cell proportions on HPV-positive and -negative head and neck squamous cell carcinoma (HNSC). Upper: HNSC-HPVpos, HPV-positive HNSC patients. Lower: HNSC-HPVneg, HPV-negative HNSC patients.
