# Supplementary material for: Bioinformatics analysis of the role of CXC ligands in the microenvironment of head and neck tumor
Source: Aging (Albany NY). 2021 Jul 11;13(13):17789–817. doi: 10.18632/aging.203269 (PMC8312447; doi:10.18632/aging.203269)
Supplement: Supplementary Tables [file aging-13-203269-s002.pdf]

SUPPLEMENTARY TABLES

Supplementary Table 1. Analysis of CXC-motif chemokine ligands (CXCLs) and chemokine receptors.

Chemokine	Other names	Official full name	Receptor
CXCL1	FSP; GRO1; GROa; MGSA; NAP-3; SCYB1; MGSA-a	C-X-C motif chemokine ligand 1	CXCR1,2
CXCL2	GRO2; GROb; MIP2; MIP2A; SCYB2; MGSA-b; MIP-2a; CINC-2a	C-X-C motif chemokine ligand 2	CXCR2
CXCL3	GRO3; GROg; MIP2B; SCYB3; MIP-2b; CINC-2b	C-X-C motif chemokine ligand 3	CXCR2,3
CXCL4	PF-4; CXCL4; SCYB4	Platelet factor 4	CXCR3
CXCL5	SCYB5; ENA-78	C-X-C motif chemokine ligand 5	CXCR1,2
CXCL6	GCP2; CKA-3; GCP-2; SCYB6	C-X-C motif chemokine ligand 6	CXCR1,2
CXCL7	PBP; TC1; TC2; TGB; LDGF; MDGF; TGB1; B-TG1; CTAP3; CXCL7; NAP-2; SCYB7; THBGB; LA-PF4; THBGB1; Beta-TG; CTAPIII; CTAP-III	Pro-platelet basic protein	CXCR2
CXCL8	IL8; NAF; GCP1; LECT; LUCT; NAP1; GCP-1; LYNAP; MDNCF; MONAP; NAP-1; SCYB8	C-X-C motif chemokine ligand 8	CXCR1,2
CXCL9	CMK; MIG; Humig; SCYB9; crg-10	C-X-C motif chemokine ligand 9	CXCR3
CXCL10	C7; IFI10; INP10; IP-10; crg-2; mob-1; SCYB10; gIP-10	C-X-C motif chemokine ligand 10	CXCR3
CXCL11	IP9; H174; IP-9; b-R1; I-TAC; SCYB11; SCYB9B	C-X-C motif chemokine ligand 11	CXCR3
CXCL12	IRH; PBSF; SDF1; TLSF; TPAR1; SCYB12	C-X-C motif chemokine ligand 12	CXCR4,7
CXCL13	BLC; BCA1; ANGIE; BCA-1; BLR1L; ANGIE2; SCYB13	C-X-C motif chemokine ligand 13	CXCR3,5
CXCL14	KEC; KS1; BMAC; BRAK; NJAC; MIP2G; MIP-2g; SCYB14	C-X-C motif chemokine ligand 14	-
CXCL16	SRPSOX; CXCLG16; SR-PSOX	C-X-C motif chemokine ligand 16	CXCR6
CXCL17	DMC; VCC1; Dcip1; VCC-1; UNQ473	C-X-C motif chemokine ligand 17	-

Supplementary Table 2. CXC-motif chemokine ligand (CXCL) transcript expression in different types of head and neck squamous cell carcinoma (HNSC) and normal tissues.

	Type of HNSC versus normal tissue	Fold change	*p*-value	*t*-test	Source and/or reference
CXCL1	Tongue Squamous Cell Carcinoma	10.936	6.96E-11	8.880	Ye Head-Neck [29]
	Tonsillar Carcinoma	6.417	3.94E-5	5.494	Pyeon Multi-cancer [30]
	Tongue Carcinoma	6.078	3.62E-7	6.027	Pyeon Multi-cancer [30]
	Head and Neck Squamous Cell Carcinoma	17.404	4.65E-10	10.508	Ginos Head-Neck [31]
	Oral Cavity Squamous Cell Carcinoma	3.891	1.35E-10	8.527	Peng Head-Neck [32]
	Tongue Squamous Cell Carcinoma	3.800	1.65E-6	5.181	Estilo Head-Neck [33]
	Tongue Squamous Cell Carcinoma	2.958	1.60E-6	5.190	Talbot Lung [34]
CXCL2	Head and Neck Squamous Cell Carcinoma	12.793	2.53E-8	8.626	Ginos Head-Neck [31]
CXCL3	Head and Neck Squamous Cell Carcinoma	5.374	4.25E-10	7.831	Ginos Head-Neck [31]
	Nasopharyngeal Carcinoma	2.328	1.90E-5	4.750	Sengupta Head-Neck [35]
CXCL5	Head and Neck Squamous Cell Carcinoma	16.431	7.42E-11	8.134	Ginos Head-Neck [31]
CXCL6	Tongue Squamous Cell Carcinoma	2.020	5.29E-5	4.504	Ye Head-Neck [29]
	Head and Neck Squamous Cell Carcinoma	2.694	4.79E-7	5.557	Ginos Head-Neck [31]
CXCL8	Tongue Squamous Cell Carcinoma	10.158	7.11E-11	9.644	Ye Head-Neck [29]
	Head and Neck Squamous Cell Carcinoma	36.405	2.12E-15	13.407	Ginos Head-Neck [31]
	Oral Cavity Squamous Cell Carcinoma	20.342	2.76E-20	14.056	Peng Head-Neck [32]
	Tongue Carcinoma	6.088	1.64E-5	5.195	Pyeon Multi-cancer [30]
CXCL9	Oral Cavity Squamous Cell Carcinoma	16.067	1.51E-18	12.430	Peng Head-Neck [32]
	Nasopharyngeal Carcinoma	5.606	3.83E-7	6.901	Sengupta Head-Neck [35]
	Head and Neck Squamous Cell Carcinoma	6.976	1.81E-10	7.860	Ginos Head-Neck [31]
	Tongue Squamous Cell Carcinoma	6.133	1.29E-6	5.279	Estilo Head-Neck [33]
CXCL10	Nasopharyngeal Carcinoma	7.729	7.60E-12	11.631	Sengupta Head-Neck [35]
	Head and Neck Squamous Cell Carcinoma	6.410	1.15E-11	8.488	Ginos Head-Neck [31]
	Oral Cavity Squamous Cell Carcinoma	19.340	6.43E-16	12.378	Peng Head-Neck [32]
	Tongue Squamous Cell Carcinoma	9.441	1.77E-7	5.885	Estilo Head-Neck [33]
	Tongue Squamous Cell Carcinoma	3.073	1.28E-6	5.738	Talbot Lung [34]
CXCL11	Nasopharyngeal Carcinoma	14.625	5.58E-10	10.466	Sengupta Head-Neck [35]
	Oral Cavity Squamous Cell Carcinoma	20.050	4.64E-17	11.661	Peng Head-Neck [32]
	Head and Neck Squamous Cell Carcinoma	6.635	3.79E-11	8.164	Ginos Head -Neck [31]
	Tongue Squamous Cell Carcinoma	7.827	4.21E-7	5.658	Estilo Head-Neck [33]
CXCL12	Oral Cavity Squamous Cell Carcinoma	-3.001	2.34E-6	-5.199	Peng Head-Neck [32]
CXCL13	Head and Neck Squamous Cell Carcinoma	67.011	3.47E-16	14.825	Ginos Head -Neck [31]
CXCL17	Nasopharyngeal Carcinoma	-4.780	1.53E-8	-7.924	Sengupta Head-Neck [35]
	Oral Cavity Squamous Cell Carcinoma	-3.444	1.14E-13	-8.939	Peng Head-Neck [32]

Supplementary Table 3. Top 50 similar CXC-motif chemokine ligand (CXCL)-related genes detected.

Gene symbol	Gene ID	PCC
GBP5	ENSG00000154451.14	0.77
EBF1	ENSG00000164330.16	0.77
CDH5	ENSG00000179776.17	0.73
FGF7	ENSG00000140285.9	0.73
BICC1	ENSG00000122870.11	0.72
CD34	ENSG00000174059.16	0.72
COL15A1	ENSG00000204291.10	0.72
CXCR2P1	ENSG00000229754.1	0.72
JAM2	ENSG00000154721.14	0.72
WARS	ENSG00000140105.17	0.72
SNED1	ENSG00000162804.13	0.71
ZEB2	ENSG00000169554.16	0.71
CD93	ENSG00000125810.9	0.70
CXorf36	ENSG00000147113.16	0.70
FBLN5	ENSG00000140092.14	0.70
GBP1	ENSG00000117228.9	0.70
IL18BP	ENSG00000137496.17	0.70
PECAM1	ENSG00000261371.5	0.70
ABCC9	ENSG00000069431.10	0.69
ARHGEF15	ENSG00000198844.10	0.69
CNRIP1	ENSG00000119865.8	0.69
FBN1	ENSG00000166147.13	0.69
FYN	ENSG00000010810.17	0.69
GPIHBP1	ENSG00000277494.1	0.69
LGALS17A	ENSG00000226025.9	0.69
PCDH12	ENSG00000113555.5	0.69
PPBP	ENSG00000163736.3	0.69
STARD8	ENSG00000130052.13	0.69
ZMYND15	ENSG00000141497.13	0.69
ADGRL4	ENSG00000162618.12	0.68
BATF2	ENSG00000168062.9	0.68
COL3A1	ENSG00000168542.12	0.68
ECM2	ENSG00000106823.12	0.68
ECSCR	ENSG00000249751.3	0.68
FZD4	ENSG00000174804.3	0.68
LAP3	ENSG00000002549.12	0.68
MYCT1	ENSG00000120279.6	0.68
S1PR1	ENSG00000170989.8	0.68
TIE1	ENSG00000066056.13	0.68
RP11	ENSG00000269998.1	0.68
COLEC12	ENSG00000158270.11	0.67
DCHS1	ENSG00000166341.7	0.67
DDR2	ENSG00000162733.16	0.67
FIBIN	ENSG00000176971.3	0.67
LDB2	ENSG00000169744.12	0.67

TMEM119	ENSG00000183160.8	0.67
ZCCHC24	ENSG00000165424.6	0.67
CH507	ENSG00000280019.1	0.67
IFITM1	ENSG00000185885.15	0.67
A2M	ENSG00000175899.14	0.66

PCC, Pearson correlation coefficient.

Supplementary Table 4. CXC-motif chemokine ligand (CXCL) expression in 22 immune cells in HNSC_GSE103322.

Gene	Cell type (major lineage)	Cell type (minor lineage)	Log2 fold change
CXCL2	CD4Tconv	CD4Tn	-1.46
	CD8Tex	CD8Tex	-1.48
	CD8T	CD8Tcm	-1.43
	Mono/macro	M1	1.49
	Plasma	Plasma	-1.44
CXCL3	Mono/macro	M1	1.58
CXCL8	Mono/macro	M1	1.95
CXCL10	Mono/macro	M1	1.38
CXCL12	CD4Tconv	CD4Tn	-1.18
	CD8Tex	CD8Tex	-1.21
	CD8T	CD8Tcm	-1.2
	Plasma	Plasma	-1.16
CXCL13	CD8Tex	CD8Tex	2.05
	CD8T	CD8Tcm	1.39
CXCL14	CD4Tconv	CD4Tn	-2.56
	CD4Tconv	CD4Tn	-2.31
	CD8Tex	CD8Tex	-2.56
	CD8T	CD8Tcm	-2.58
	Mast	Mast	-1.73
	Mono/macro	M1	-2.36
	Plasma	Plasma	-2.45
CXCL16	Mono/macro	M1	1.41

CD4Tconv, conventional T-cells; CD4Tn, naive T cells; CD8Tex, exhausted CD8(+) T cells; CD8Tcm, central memory CD8(+) T cells; Mono/macro, monocytes/macrophages; M1, classically activated macrophages.

Supplementary Table 5. CXC-motif chemokine ligand (CXCL) expression in 40 immune cells in HNSC_GSE139324.

Gene	Cell type (major lineage)	Cell type (minor lineage)	Log2 fold change
CXCL1	Mono/macro	Monocyte	1.91
	Mono/macro	M2	1.13
CXCL2	Mono/macro	Monocyte	2.77
	Mono/macro	M2	1.18
CXCL3	Mono/macro	Monocyte	2.7
	Mono/macro	M2	1.16
CXCL8	B	B	-1.41
	B	B	-1.48
	B	B	-1.46
	CD4Tconv	CD4Tn	-1.37
	CD4Tconv	CD4Tn	-1.59
	CD4Tconv	Tfh	-1.1
	CD8T	CD8Teff	-1.48
	CD8T	CD8Tem	-1.49
	CD8Tex	CD8Tex	-1.13
	CD8T	CD8Tn	-1.53
	Mono/macro	Monocyte	3.62
	Mono/macro	M1	-1.25
	Mono/macro	M2	1.25
	NK	NK	-1.48
CXCL9	Mono/macro	M2	1.76
CXCL10	Mono/macro	cDC2	1.13
	Mono/macro	Monocyte	2.27
	Mono/macro	M2	1.71
CXCL13	B	B	-1.24
	B	B	-1.22
	B	B	-1.21
	CD4Tconv	CD4Tn	-1.23
	CD4Tconv	CD4Tn	-1.35
	CD8T	CD8Teff	-1.28
	CD8Tex	CD8Tex	1.45
	CD8T	CD8Tem	-1.29
	CD8T	CD8Tn	-1.3
	Mono/macro	Monocyte	-1.23
	Mono/macro	Monocyte	-1.32
	Mono/macro	M1	-1.29
	NK	NK	-1.3
	Tprolif	Tprolif	1.6
	Treg	Treg	1.91
CXCL16	Mono/Macro	M2	1.23

Mono/macro, monocytes/macrophages; M1, classically activated macrophages; M2, alternatively activated macrophages; B, B cells; CD4Tconv, CD4(+) conventional T-cells; CD4Tn, CD4(+) naive T cells; Tfh, T follicular helper cells; CD8Teff, CD8(+) effector T cells; CD8Tem, CD8(+) T effector memory cells; CD8Tex, exhausted CD8(+) T cells; CD8Tn, CD8(+) naive T cells; NK, natural killer cells; cDC2, conventional dendritic cells type 2; Tprolif, proliferating T cells; Treg, regulatory T cells.

Supplementary Table 6. CXC-motif chemokine ligands (CXCLs) and immune cell infiltration in head and neck squamous cell carcinoma (HNSC) patients.

	Purity		B cells		CD8+ T cells		CD4+ T cells		Macrophages		Neutrophils		Dendritic cells	
	cor	*p*-value	cor	*p*-value	cor	*p*-value	cor	*p*-value	cor	*p*-value	cor	*p*-value	cor	*p*-value
CXCL1	-0.121	7.08E-03	-0.011	8.16E-01	0.047	3.07E-01	0.001	9.74E-01	-0.076	9.58E-02	0.086	6.04E-02	0.013	7.80E-01
CXCL2	-0.16	3.76E-04	0.016	7.24E-01	0.079	8.63E-02	0.072	1.15E-01	0.083	6.92E-02	0.194	1.93E-05	0.144	1.57E-03
CXCL3	-0.056	2.18E-01	-0.003	9.41E-01	0.088	5.45E-02	0.08	7.95E-02	0.049	2.86E-01	0.228	4.32E-07	0.159	4.75E-04
CXCL4	-0.081	7.28E-02	0.046	3.12E-01	0.048	2.93E-01	-0.017	7.09E-01	0.062	1.72E-01	0.003	9.43E-01	0.044	3.33E-01
CXCL5	-0.032	4.81E-01	-0.062	1.79E-01	-0.055	2.30E-01	-0.013	7.83E-01	0.021	6.41E-01	0.025	5.88E-01	0.048	3.13E-01
CXCL6	-0.135	2.61E-03	0.085	6.23E-02	0.057	2.17E-01	0.069	1.33E-01	0.058	2.02E-01	0.047	3.07E-01	0.047	3.07E-01
CXCL7	-0.116	1.00E-02	-0.137	2.79E-03	-0.076	9.78E-02	-0.128	5.04E-03	-0.132	3.53E-03	-0.121	8.14E-03	-0.09	4.73E-02
CXCL8	-0.015	7.40E-01	-0.106	2.11E-02	-0.032	4.92E-01	0.015	7.47E-01	-0.063	1.69E-01	0.068	1.39E-01	0.053	2.45E-01
CXCL9	-0.315	8.53E-13	0.252	2.66E-08	0.558	4.45E-40	0.524	3.60E-35	0.444	9.81E-25	0.728	4.01E-80	0.667	2.33E-63
CXCL10	-0.257	6.74E-09	0.105	2.20E-02	0.413	5.66E-21	0.415	2.05E-21	0.252	1.93E-08	0.748	5.89E-87	0.539	1.25E-37
CXCL11	-0.282	1.90E-10	0.022	6.29E-01	0.341	2.21E-14	0.365	1.41E-16	0.214	2.01E-06	0.705	2.98E-73	0.48	4.08E-29
CXCL12	-0.273	6.73E-10	0.346	8.27E-15	0.289	1.46E-10	0.514	1.15E-33	0.563	1.07E-41	0.33	1.14E-13	0.488	3.57E-30
CXCL13	-0.328	7.81E-14	0.433	3.66E-23	0.59	8.55E-46	0.495	5.48E-31	0.518	1.89E-34	0.566	6.00E-42	0.626	9.24E-54
CXCL14	-0.162	3.10E-04	-0.13	4.48E-03	-0.263	5.81E-09	-0.119	9.01E-03	-0.192	2.08E-05	-0.2	1.10E-05	-0.23	3.21E-07
CXCL16	-0.085	5.95E-02	0.291	1.03E-10	0.369	9.10E-17	0.497	2.45E-31	0.455	4.27E-26	0.497	2.69E-31	0.566	3.37E-42
CXCL17	-0.078	8.47E-02	0.157	5.96E-04	0.184	5.50E-05	0.1	2.78E-02	-0.008	8.68E-01	0	9.94E-01	0.002	9.63E-01

cor, correlation.
